# Trends in Resource Utilization by Children with Neurological Impairment in the United States Inpatient Health Care System: A Repeat Cross-Sectional Study

**DOI:** 10.1371/journal.pmed.1001158

**Published:** 2012-01-17

**Authors:** Jay G. Berry, Annapurna Poduri, Joshua L. Bonkowsky, Jing Zhou, Dionne A. Graham, Chelsea Welch, Heather Putney, Rajendu Srivastava

**Affiliations:** 1 Complex Care Service, Children's Hospital Boston, Harvard Medical School, Boston, Massachusetts, United States of America; 2 Department of Pediatric Neurology, Children's Hospital Boston, Harvard Medical School, Boston, Massachusetts, United States of America; 3 Division of Pediatric Neurology, Department of Pediatrics, University of Utah School of Medicine, Salt Lake City, Utah, United States of America; 4 Clinical Research Program, Children's Hospital Boston, Harvard Medical School, Boston, Massachusetts, United States of America; 5 Division of Inpatient Medicine, Department of Pediatrics, University of Utah School of Medicine, Salt Lake City, Utah, United States of America; 6 Institute for Community Inclusion, Boston, Massachusetts, United States of America; University of Queensland, Australia

## Abstract

**Background:**

Care advances in the United States (US) have led to improved survival of children with neurological impairment (NI). Children with NI may account for an increasing proportion of hospital resources. However, this assumption has not been tested at a national level.

**Methods and Findings:**

We conducted a study of 25,747,016 US hospitalizations of children recorded in the Kids' Inpatient Database (years 1997, 2000, 2003, and 2006). Children with NI were identified with *International Classification of Diseases, 9th Revision, Clinical Modification* diagnoses resulting in functional and/or intellectual impairment. We assessed trends in inpatient resource utilization for children with NI with a Mantel-Haenszel chi-square test using all 4 y of data combined. Across the 4 y combined, children with NI accounted for 5.2% (1,338,590) of all hospitalizations. Epilepsy (52.2% [*n* = 538,978]) and cerebral palsy (15.9% [*n* = 164,665]) were the most prevalent NI diagnoses. The proportion of hospitalizations attributable to children with NI did not change significantly (*p* = 0.32) over time. In 2006, children with NI accounted for 5.3% (*n* = 345,621) of all hospitalizations, 13.9% (*n* = 3.4 million) of bed days, and 21.6% (US$17.7 billion) of all hospital charges within all hospitals. Over time, the proportion of hospitalizations attributable to children with NI decreased within non-children's hospitals (3.0% [*n* = 146,324] in 1997 to 2.5% [*n* = 113,097] in 2006, *p*<.001) and increased within children's hospitals (11.7% [*n* = 179,324] in 1997 to 13.5% [*n* = 209,708] in 2006, *p*<0.001). In 2006, children with NI accounted for 24.7% (2.1 million) of bed days and 29.0% (US$12.0 billion) of hospital charges within children's hospitals.

**Conclusions:**

Children with NI account for a substantial proportion of inpatient resources utilized in the US. Their impact is growing within children's hospitals. We must ensure that the current health care system is staffed, educated, and equipped to serve this growing segment of vulnerable children.

*Please see later in the article for the Editors' Summary*

## Abstract 2

Jay Berry and colleagues report findings from an analysis of hospitalization data in the US, examining the proportion of inpatient resources attributable to care for children with neurological impairment.

## Editors' Summary

**Background:**

Disorders of the central and peripheral nervous system, often referred to as neurological impairments, are common in infants and children and can cause functional or intellectual disability. There are many causes of neurological impairments, including birth trauma, congenital abnormalities, structural defects, infections, tumors, blood flow disruption, genetic and metabolic conditions, and toxins. Symptoms can be progressive or static and vary widely depending on the condition. For example, developmental delay, changes in activity—often due to muscle wasting—and seizures may be common symptoms of neurological conditions in children. In many countries, extremely premature babies, and children with conditions such as spina bifida and muscular dystrophy, now receive better care than they used to, and may survive longer. However, although such children may have long-term care needs, they may receive crisis-driven, uncoordinated care, even in high-income countries.

**Why Was This Study Done?:**

It is not well understood what proportion of hospital resource use is attributable to care for children with neurological impairments, although it's thought that this group may account for an increasing proportion of hospital resources. In this study, the researchers attempted to answer this question, specifically for the US, by evaluating national trends in hospital admissions for children with neurological impairments.

**What Did the Researchers Do and Find?:**

The researchers used a multi-state database of US hospital admissions for children aged 0–18 years, known as the KID—the Healthcare Cost and Utilization Project's Kids' Inpatient Database—to identify the number of hospital admissions, total number of days spent in the hospital, and total health care costs for children with neurological impairments from 1997 to 2006. The researchers identified appropriate admissions by using diagnostic codes from the *International Classification of Diseases, 9th Revision, Clinical Modification* (ICD-9-CM), which were reviewed and approved by two pediatric neurologists.

The researchers found that from 1997 to 2006, there were 25,747,016 hospital admissions for children aged 0–18 years, and of these, 1,338,590 (5.2%) were associated with children who had a definite diagnosis of neurological impairment. The most prevalent diagnoses among all hospitalized children with neurological impairments were epilepsy (52.2%) and cerebral palsy (15.9%). Furthermore, across the study period, the proportion of children aged 13–18 years admitted to hospitals with neurological impairments increased from 7.3% to 9.9%. The researchers also found that children with neurological impairments accounted for an increasing proportion of days spent in a hospital (12.9% in 1997 to 13.9% in 2006). In addition, there was a substantial increase in admissions for infants with neurological impairments compared to infants without neurological impairments. The researchers also found that throughout the study period, there was a general pattern for children with neurological impairments to be admitted to pediatric hospitals, rather than general hospitals. Within children's hospitals, children with neurological impairments accounted for a substantial proportion of resources over the study period, including nearly one-third of all hospital charges.

**What Do These Findings Mean?:**

These findings show that in the US, children with neurological impairments account for a substantial proportion of inpatient resources utilized, particularly within children's hospitals, necessitating the need for adequate clinical care and a coordination of efforts to ensure that the needs of children with neurological impairments are met. System-based efforts such as partnerships between hospitals and families of children with neurological impairments and the rigorous evaluation of care treatment strategies have the potential to promote quality care for children with neurological impairments. However, such efforts will work only if the current health care system is adequately staffed with appropriately educated professionals.

**Additional Information:**

Please access these websites via the online version of this summary at http://dx.doi.org/pmed.1001158.

- More information is available about the KID database used in this study
- NHS Choices has further information about epilepsy, one of the most common types of neurological impairment examined in this study
- Further information is available from PubMed Health about cerebral palsy, another neurological condition acquired during development that was studied in this dataset

## Introduction

Neurological impairment (NI) comprises a heterogeneous group of static and progressive health conditions that involve the central and peripheral nervous systems and result in functional and/or intellectual impairment. This group includes children with epilepsy, infants who are born prematurely and have hypoxic-ischemic injury to the brain, and children with genetic and metabolic disorders that affect the nervous system.

Although the impact that children with NI have on the pediatric health care system remains largely unknown, emerging evidence describes epidemiologic and care delivery phenomena that suggest this impact may be substantial. The rising prevalence and improved survival of extremely premature infants was associated with an increased risk of cerebral palsy during the 1990s [1],[2]. Children with spina bifida, muscular dystrophy, and other NI-related diagnoses are also surviving longer [3],[4], in part because of improved co-morbid condition management of gastroesophageal reflux disease, oromotor dysfunction, and chronic lung disease [5],[6].

In the US, many children with NI receive uncoordinated, crisis-driven care that is believed to contribute to excessive health care utilization and cost [7]–[11]. They have a disproportionately high use of emergency care health services, and they experience frequent hospitalizations, with subsequently high readmission rates [12],[13]. When hospitalized, they tend to stay longer and have more expensive admissions than other children [14]. It is estimated that a large proportion of lifetime health-related costs for a children with severe NI is attributable to inpatient care [15].

This evidence and our clinical experience suggest that children with NI may account for an increasing proportion of hospital resources. However, this assumption has not been tested at a national level for the US. We undertook this study to (1) evaluate national trends in hospitalizations for children with NI over time within different types of hospitals and (2) describe the patient characteristics and reasons why children with NI are hospitalized.

## Methods

### Ethics Statement

This study was approved by the Children's Hospital Boston Institutional Review Board with a waiver for informed consent. The investigation was conducted according to the principles expressed in the Declaration of Helsinki.

### Study Design and Setting

This is a retrospective, repeat cross-sectional analysis of the Healthcare Cost and Utilization Project Kids' Inpatient Database (KID) for the years 1997, 2000, 2003, and 2006. The KID is a multi-state database of US hospitalizations for children aged 0–18 y [16],[17]. From 1997 to 2006, the KID sample increased from 1.9 million hospital discharges from 2,521 hospitals in 22 states to 3.1 million hospital discharges from 3,739 hospitals in 38 states [16],[17]. The dataset includes a weight variable for each observation that was used to produce national estimates of inpatient resource utilization for children with specific diagnoses while accounting for differences in the KID hospital sampling frame and oversampling of patient sub-populations that could have occurred over time.

### Study Population

We identified hospitalizations for children aged 0–18 y with NI as determined by *International Classification of Diseases, 9th Revision, Clinical Modification* (ICD-9-CM) diagnostic codes. Up to 15 separate ICD-9-CM diagnostic codes are available for each hospitalization in the KID [17]. We assembled an initial list of 606 NI ICD-9-CM codes from previous studies and the tabular index of ICD-9-CM coding [18]–[20].

#### Pediatric neurologist review of NI ICD-9-CM codes

**Figure 1:**
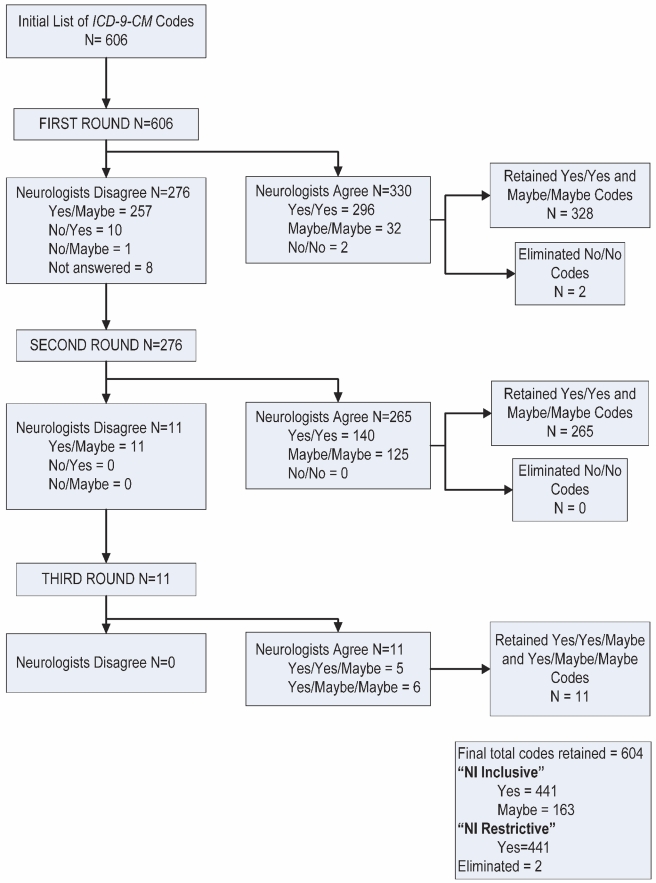
Neurological impairment diagnosis code evaluation. This figure describes the results of the NI diagnosis code evaluation by independent pediatric neurologists.

Two board-certified pediatric neurologists (A. P. and J. L. B.) from different institutions reviewed the initial NI code list [21]. They considered candidate NI ICD-9-CM codes using the following definition: “diagnosis is consistent with NI (static or progressive) and typically results in either functional and/or intellectual impairment” (Figure 1) [19],[20]. The reviewers independently classified each code as “yes,” (diagnosis consistent with NI), “no” (diagnosis not consistent with NI), or “maybe” (diagnosis may be consistent with NI). Code consensus was defined as classification agreement between the reviewers. The reviewers then discussed the codes for which there was classification disagreement (*n* = 276 [46%]) until consensus was reached (*n* = 265 [96%]) or was not reached (*n* = 11 [4%]). A third pediatric neurologist (F. Filloux) classified the remaining non-consensus codes.

For the main analyses, we retained the NI codes classified “yes–yes” (*n* = 436), “yes–maybe” (*n* = 5), and “maybe–maybe” (*n* = 163). We labeled the codes “NI inclusive.” For the subsequent sensitivity analysis, we excluded the “maybe–maybe” codes from analysis and labeled the retained codes “NI restricted” (Figure 1).

The NI restricted codes were then compared to a list describing coding changes over time assembled by the National Center for Health Statistics and the Centers for Medicare and Medicaid Services. Eleven NI ICD-9-CM codes changed from 1997 to 2006. We incorporated these code changes into our analysis. NI-related hospitalizations were identified from the presence of one or more NI ICD-9-CM codes in any of the discharge diagnoses. The NI ICD-9-CM codes are presented in Table S1.

### Main Outcomes

We evaluated the number of hospitalizations, total number of days spent in the hospital, and total charges for admissions associated with the NI inclusive and NI restricted admissions for each year.

#### Hospital type

We analyzed the measures of NI inpatient resource utilization within all hospitals, children's hospitals, and non-children's hospitals [16],[17]. “Children's hospital” designation was given to hospitals based on specifications from the National Association of Children's Hospitals and Related Institutions. Children's hospitals included freestanding children's hospitals, children's hospitals within adult general hospitals, and children's specialty hospitals.

#### Reasons for hospitalization

We evaluated patient demographic and clinical characteristics that may correlate with trends in hospital resource use for children with NI over time. Patient demographic variables included patient age (<1 y, 1–4 y, 5–12 y, and 13–18 y) and insurance type (public, private, and other). Race/ethnicity was described but not analyzed because of the extent of missing data (17%–28% for the years under study).

We also analyzed each NI hospitalization to determine if an NI-related surgical procedure occurred. These procedures were classified based on ICD-9-CM procedure codes from previous literature on surgical procedures that these children frequently encounter [6],[14],[20],[22],[23]. We specifically assessed hospitalizations where a gastrostomy (codes beginning with 43.1 or 44.32) and/or fundoplication (codes 44.66 or 44.67) occurred.

We analyzed the principal reason for each hospitalization in children with NI based on the ICD-9-CM Major Diagnostic Category (MDC). MDCs represent 25 mutually exclusive diagnosis domains primarily organized by organ system (nervous system, respiratory system, circulatory system, etc.). One MDC is assigned for each hospitalization from the patient's principal ICD-9-CM diagnosis code. The “newborns and other neonates” MDC includes newborns cared for in a neonatal intensive care unit or in a healthy newborn unit of a maternity hospital.

### Statistical Analyses

We evaluated national inpatient resource utilization trends for children with and without NI using all of the years of KID data with a Mantel-Haenszel chi-square test. We used SAS version 9.1.2 (SAS Institute) survey procedures to account for the changing KID sampling frame through a weight variable calculated from the demographic characteristics of the hospitals contributing to the database (ownership/control, bed size, teaching status, children's hospital status, rural/urban location, and state/region) and their hospitalized patients. As the sampling frame expanded over time, the discharge weight decreased so that the increasing proportion of actual records did not falsely inflate the national estimates [24]. All patient-level data were clustered within each hospital and stratified by the demographic characteristics of each hospital to generate national estimates and associated variances of the proportion of inpatient resource utilization attributable to children with NI. Significance threshold was defined as *p*≤0.05. We adjusted total hospital charges for inflation to 2006 US dollars [25],[26].

## Results

**Figure 2:**
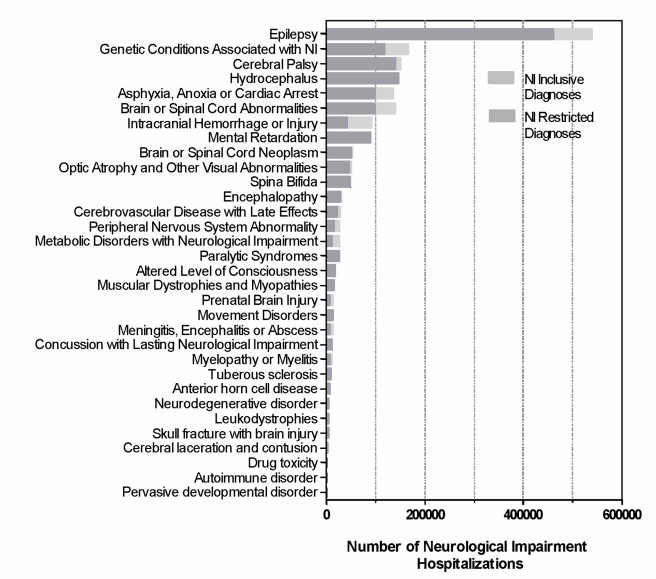
Neurological impairment diagnoses of hospitalized children, Kids' Inpatient Database 1997, 2000, 2003, and 2006. This graph describes the proportion of total NI hospitalizations attributable to each of the NI diagnosis categories.

There were 25,747,016 hospitalizations for children aged 0–18 y identified from 1997, 2000, 2003, and 2006 combined. Of these, 1,338,590 (5.2%) hospitalizations were associated with children who had NI inclusive diagnoses, and 1,032,829 (4.0%) hospitalizations were associated with children who had NI restricted diagnoses (i.e., the NI codes classified as “maybe–maybe” were excluded). NI inclusive results are presented throughout the Results, with NI restricted results displayed in the table and figures. The most prevalent diagnoses among all hospitalized children with NI were epilepsy (52.2% [*n* = 538,978]) and cerebral palsy (15.9% [*n* = 164,665]) (Figure 2). The NI diagnoses varied by age: cerebral palsy was diagnosed in 1.1% (*n* = 4,708) of hospitalized infants with NI, compared with 17.4% (*n* = 102,590) of children aged 13–18 y.

Throughout the study period the proportion of hospitalized children aged 13–18 y that had NI increased from 7.3% to 9.9% (*p*<0.001) (Table 1). Children with NI aged 13–18 y had a greater increase in hospitalizations (27.8% increase [52,459 in 1997 to 67,045 in 2006]) than children without NI (8.4% decrease [666,403 in 1997 to 610,246 in 2006]). Similar demographic results were observed in children with an NI restricted diagnosis and in children utilizing children's hospitals and non-children's hospitals (data not shown).

**Table 1:** Demographic data for children hospitalized with neurological impairment, Kids' Inpatient Database.

Characteristics	Patients	Year				*p*-Value^a^
		1997	2000	2003	2006	
**Number of hospitalizations**	All children	6,348,671	6,351,352	6,468,925	6,578,068	
	Percent NI	5.2% (4.0%)	5.0% (3.9%)	5.4% (4.2%)	5.3% (3.9%)	0.32
**Age at admission**						
<1 y	All children	4,437,620	4,539,180	4,588,429	4,761,374	
	Percent NI	2.4% (1.5%)	2.3% (1.4%)	2.4% (1.5%)	2.4% (1.4%)	0.41
1–5 y	All children	611,283	542,439	598,562	572,808	
	Percent NI	14.4% (12.3%)	13.7% (11.4%)	13.7% (11.5%)	13.1% (10.3%)	0.08
5–12 y	All children	585,078	577,687	609,384	566,596	
	Percent NI	14.1% (12.1%)	15.0% (12.9%)	15.3% (13.0%)	16.2% (13.3%)	0.001
13–18 y	All children	718,862	692,045	672,549	677,291	
	Percent NI	7.3% (6.3%)	8.1% (7.0%)	9.2% (7.9%)	9.9% (8.4%)	<0.001
**Insurance type**						
Public	All children	2,330,189	2,334,780	2,662,043	2,916,538	
	Percent NI	6.2% (4.9%)	5.9% (4.7%)	6.0% (4.8%)	5.8% (4.4%)	0.62
Private	All children	3,410,217	3,501,436	3,288,596	3,124,969	
	Percent NI	4.6% (3.6%)	4.5% (3.5%)	4.9% (3.7%)	4.8% (3.5%)	0.55
Other	All children	349,394	311,447	294,484	329,858	
	Percent NI	3.4% (2.6%)	3.0% (2.2%)	3.3% (2.4%)	3.0% (2.0%)	0.28
**Female gender**	All children	3,113,005	3,117,038	3,137,454	3,201,426	
	Percent NI	4.7% (3.7%)	4.5% (3.5%)	4.9% (3.8%)	4.9% (3.6%)	0.40
**Race/ethnicity**						
Non-Hispanic white	All Children	2,721,511	2,984,746	2,416,538	2,481,431	
	Percent NI	5.1% (4.0%)	5.2% (4.1%)	5.6% (4.3%)	5.5% (4.1%)	—b
Non-Hispanic black	All Children	810,682	780,618	680,395	705,283	
	Percent NI	5.9% (4.7%)	5.7% (4.5%)	6.3% (5.0%)	6.0% (4.5%)	—b
Hispanic	All children	750,077	1,053,009	1,100,883	1,184,642	
	Percent NI	5.2% (4.1%)	4.8% (3.7%)	4.9% (3.8%)	4.8% (3.6%)	—b
Other	All children	325,002	449,074	462,284	480,807	
	Percent NI	5.1% (3.8%)	4.8% (3.6%)	5.0% (3.8%)	5.1% (3.7%)	—b
Missing	All children	1,745,270	1,083,368	1,807,044	1,725,358	
	Percent NI	5.0% (3.9%)	4.6% (3.5%)	5.2% (4.0%)	4.9% (3.6%)	—b

Percentages are NI inclusive values, with NI restricted values given in parentheses.

a *p*-Value of a Mantel-Haenszel chi-square test for all patients with NI.

b Race/ethnicity testing not performed because of the extent of missing data.

### All Hospitals

#### Inpatient resource utilization

**Figure 3:**
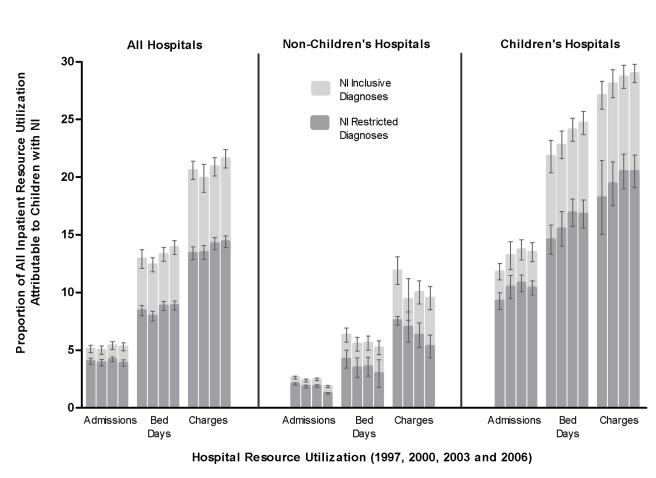
Inpatient resource utilization attributable to children with neurological impairment, Kids' Inpatient Database 1997, 2000, 2003, and 2006, by hospital type. These graphs describe the proportion of all pediatric inpatient health resources attributable to NI from each year. Proportion (with 95% confidence interval) of total number of hospitalizations, hospital bed days, and total aggregate charges are shown for all hospitals, non-children's hospitals, and children's hospitals. Each bar represents 1 y of data. The four bars in each group represent data from 1997, 2000, 2003, and 2006.

There was no significant change in the proportion of hospitalizations attributable to children with NI (5.1% [*n* = 325,556] in 1997 to 5.3% [*n* = 345,621] in 2006, *p* = 0.32) or the proportion of total charges (20.8% [US$9.8 billion] in 1997 to 21.6% [US$17.7 billion] in 2006, *p* = 0.09) (Figure 3). However, children with NI accounted for an increasing proportion of hospital days (12.9% in 1997 to 13.9% in 2006, *p* = 0.05). They experienced a greater increase in hospital days (22.2% increase [2.8 million in 1997 to 3.4 million in 2006]) than children without NI (11.6% increase [18.9 million in 1997 to 21.1 million in 2006], *p* = 0.05). Similar patterns were observed for children with NI restricted diagnoses.

#### Reasons for admission

There was a greater increase in admissions for infants with NI (15.6% increase [57,147 in 1997 to 66,046 in 2006]) than for infants without NI (9.9% increase [3,832,736 in 1997 to 4,212,576 in 2006], *p*<0.001). There were opposing trends in neurological admissions for children with NI (7.0% increase [105,911 in 1997 to 113,321 in 2006]) and children without NI (8.1% decrease [65,731 in 1997 to 60,437 in 2006], *p*<0.001). There was a greater decrease in respiratory admissions for children with NI (20.5% decrease [48,334 in 1997 to 38,390 in 2006]) than children without NI (13.2% decrease [546,111 in 1997 to 474,125 in 2006], *p*<0.001). There was an increase in the number of gastrostomy/fundoplication procedures that was similar for children with NI (27.1% increase [7,579 in 1997 to 9,638 in 2006]) and children without NI (23.7% increase [6,507 in 1997 to 8,048 in 2006], *p* = 0.5).

In 2006, the most common reasons for admission in children with NI were for neonatal care (19.1%, *n* = 66,046), seizures (16.9%, *n* = 58,253), or other neurologic (13.6%, *n* = 46,932) or respiratory (11.1%, *n* = 38,390) problems.

### Non-Children's Hospitals

#### Inpatient resource utilization

Within non-children's hospitals, children with NI accounted for a decreasing proportion of hospitalizations (3.0% in 1997 to 2.5% in 2006, *p*<0.001]) (Figure 3). Children with NI experienced a decreasing admission trend (22.7% decrease [146,232 in 1997 to 113,097 in 2006]) that was greater than that for children without NI (4.2% decrease [4,678,507 in 1997 to 4,483,695 in 2006], *p*<0.001). Children with NI accounted for a decreasing proportion of hospital charges (14.2% in 1997 to 13.0% in 2006, *p*<0.001). They experienced an increase in charges (39.1% increase [US$3.4 billion in 1997 to US$4.7 billion in 2006]) that was less than the increase for children without NI (47.1% increase [US$20.6 billion in 1997 to US$30.3 billion in 2006], *p*<0.001). There was no significant change in the proportion of bed days attributable to children with NI (8.0% [*n* = 1.1 million] in 1997 versus 7.7% [*n* = 1.1 million] in 2006, *p* = 0.62). Similar patterns were observed in patients with an NI restricted diagnosis.

#### Reasons for admission

Within non-children's hospitals, there was a greater increase in admissions for infants with NI (8.1% increase [35,544 in 1997 to 38,425 in 2006]) than for infants without NI (3.8% increase [3,272,710 in 1997 to 3,395,539 in 2006], *p*<0.001). There was a decreasing neurological admissions trend that was greater in children with NI (33.1% decrease [40,968 in 1997 to 27,386 in 2006]) than in children without NI (25.9% decrease [34,957 in 1997 to 25,889 in 2006], *p*<0.001). There was also a decreasing respiratory admissions trend that was greater in children with NI (50.1% decrease [25,802 in 1997 to 12,882 in 2006]) than in children without NI (24.9% decrease [374,151 in 1997 to 280,729 in 2006], *p*<0.001).

In 2006, neonatal admissions accounted for 33.9% (*n* = 38,425) of all NI pediatric admissions, 65.7% (*n* = 727,465) of NI hospital days, and 62.7% (US$2.9 billion) of NI hospital charges within non-children's hospitals.

### Children's Hospitals

#### Inpatient resource utilization

Throughout the study period, children with NI accounted for an increasing proportion of hospitalizations (11.7% in 1997 to 13.5% in 2006, *p*<0.001) within children's hospitals (Figure 3). They experienced a greater increase in admissions (16.9% increase [179,324 in 1997 to 209,708 in 2006]) than children without NI (0.2% increase [1,344,608 in 1997 to 1,347,041 in 2006], *p*<0.001). Children with NI accounted for an increasing proportion of bed days (21.8% in 1997 to 25.0% in 2006, *p*<0.001). They experienced a greater increase in bed days (22.0% increase [1.7 million in 1997 to 2.1 million in 2006]) than children without NI (4.0% increase [6.1 million in 1997 to 6.3 million in 2006], *p*<0.001). Children with NI accounted for an increasing proportion of hospital charges (27.1% in 1997 to 29.0% in 2006, *p*<0.001). They experienced a greater increase in hospital charges (84.2% increase [US$6.5 to $12.0 billion]) than children without NI (67.3% increase [US$17.4 billion to 29.2 billion], *p*<0.001). Similar patterns were observed in patients with a NI restricted diagnosis (Figure 3).

#### Reasons for admission

Within children's hospitals, there were opposing trends for neonatal admissions in infants with NI (5.0% increase [21,799 in 1997 to 22,882 in 2006]) and infants without NI (1.5% decrease [560,026 in 1997 to 551,807 in 2006], *p*<0.001). There was an increasing neurological admissions trend for children with NI (25.2% increase [63,234 in 1997 to 79,232 in 2006]) that was not observed in children without NI (0.0% increase [30,774 in 1997 to 30,630 in 2006], *p*<0.001). There were opposing trends observed in respiratory admissions for children with NI (1.2% increase [22,686 in 1997 to 22,958 in 2006]) versus for children without NI (6.4% decrease [171,959 in 1997 to 161,026 in 2006], *p*<0.001). Similar results were observed for children with NI restricted diagnoses.

In 2006, the most common reasons for admission of children with NI within children's hospitals were for seizures (17.8%, *n* = 37,246), other neurologic problems (20.0%, *n* = 41,986), respiratory problems (10.9%, *n* = 22,958), or neonatal care (10.9%, *n* = 22,883).

## Discussion

The main findings from this study suggest that inpatient resource utilization for children with NI in the US has changed from 1997 to 2006. The changes were largely attributable to children with NI utilizing children's hospitals more over time. Within children's hospitals, they accounted for both increasing trends and a substantial proportion of resources over the study period, including nearly one-third of all hospital charges. Within all hospitals, infants and children with NI experienced a greater increase in neonatal and neurological admissions and a greater decrease in respiratory admissions than children without NI. The majority of admissions for children with NI occurred for non-nervous-system problems, suggesting that co-morbid conditions affecting other organ systems were a major contributor to inpatient utilization.

Our study contributes to existing literature by describing national inpatient utilization for children with NI who had underlying primary neurological diagnoses (e.g., cerebral palsy) or other diagnoses that are characteristically associated with co-morbid nervous system impairment (e.g., Down Syndrome). As expected, studies restricted to a cohort of children with primary neuromuscular diagnoses report less inpatient utilization (e.g., 2% of all pediatric admissions and 7% of all pediatric hospital charges) than the present study [27]. Our findings are consistent with adult studies reporting that NI associated with intellectual disabilities and dementia ranks among the top determinants of health care cost [28],[29]. Although the underlying diagnoses and reasons for NI may be different across the age spectrum, patients with NI may account for a substantial share of both pediatric and adult health care resources.

There is a rising proportion of children's hospital use by children with NI, as the total number of hospitalizations for children without NI did not increase within children's hospitals over time. Most US child neurologists work within children's hospitals, and there has been an emergence of multi-disciplinary care coordination clinics that provide comprehensive care for children with NI at children's hospitals [30],[31]. Children with NI may be utilizing children's hospitals more often to seek care from providers who are more familiar with and comfortable managing their health problems. Further study is necessary to evaluate whether clinician NI knowledge and care delivery differ between children's and non-children's hospitals.

US physicians have demonstrated deficiencies in working knowledge of the most common forms of NI, and negative provider attitudes toward this population are associated with substandard acute care practices [32],[33]. Parents of adolescents with NI report this as a contributing factor that complicates the transfer of their child's health care from pediatric to adult providers [34]. In the present study, we observed a trend of rising hospitalizations in adolescent children with NI that was greater than that for children without NI. Further investigation is necessary to determine whether hindered care transition is contributing to this trend.

The rise in the number of admissions associated with gastrostomy with and without fundoplication operations in children with and without NI over time may represent a change in approach to more interventions to bypass oromotor dysfunction, mitigate aspiration from oral feeds, and improve gastroesophageal reflux management in these children. The appropriateness and effectiveness of these operations in children with NI are currently under investigation. It is possible that the decrease in the absolute number of respiratory admissions for children with NI could be related to the rise in gastrostomy and/or fundoplication operations: future studies should investigate whether these operations help prevent pneumonia admissions due to reflux and aspiration [35]. We were unable to evaluate this directly in the present study, as the KID does not track individual patients before and after operations.

In the present study, the absolute number of respiratory admissions decreased for children both with and without NI. Prior small area studies report that children with intellectual disability are at higher risk for respiratory hospitalizations that may be ambulatory care sensitive than children without intellectual disability [36]. Our study suggests that, nationally, children with NI may have received an equivalent or greater benefit from efforts to improve respiratory heath in children, including better respiratory preventive measures and acute and chronic respiratory care treatments [37],[38]. It is possible that some children with NI could have gained exposure to these efforts through improved care coordination within medical homes or other structured clinical programs [31].

The increasing trend of neonatal admissions for infants with NI is supported by prior studies that report a rising incidence of NI associated with increasing preterm infant deliveries and preterm infant survival [1]. However, some recent studies show that the neonatal prevalence of certain NI-related diagnoses, such as cerebral palsy, is now decreasing [39]. This may be related to perinatal care improvements that minimize the risk of brain injury, as well as to changing practices associated with prenatal congenital malformation screening and elective termination [40]. Using inpatient administrative billing codes to detect NI among neonates limits the interpretation of the trends observed in the present study. Some infants may not be diagnosed with NI until well after their neonatal hospitalization.

Our study has several other limitations. We were unable to determine whether the trends could have been a function of the changing population prevalence of children with NI at large in the US. US census data indicate that the population of all children aged 0–18 y increased by 5.6% from 1997 to 2006. However, to our knowledge, true population prevalence trends of all children with NI remain unknown.

The large shift in the inclusion of hospitals from different states in the KID sampling frame could have affected the study findings. In a post hoc analysis, we restricted the cohort to patients admitted to hospitals that were present in both the 1997 and 2006 KID samples. Similar results were observed. The KID contains hospital discharge rather than individual patient data, which prevented us from being able to determine whether the same sized population of children with NI was being hospitalized more frequently over time. This will be important to evaluate in future health resource utilization and epidemiologic studies of children with NI.

Continuous years of data may be preferable for studying hospital utilization trends when compared with four discrete time points in a 10-y period. The hospitals within the KID used ICD-9-CM coding for all the years of the study. It will be important to evaluate the identification of NI diseases using ICD-10 and other coding systems, as many countries are currently using or implementing them. Errors in ICD-9-CM coding exist, and billing reimbursement may influence which codes are entered for a patient during hospitalization. In some cases, NI may be regarded as a subjective medical condition that cannot be correlated with a specific diagnosis code. As such, the hospital resource use described in the present study may underrepresent children with NI who are hospitalized with that situation. The KID contains hospital charge data, but not cost data. Charge data represent the amount that hospitals billed for services but does not reflect how much hospital services actually cost or the specific amounts that hospitals received in payment.

Despite these limitations, this study demonstrates that children with NI account for a substantial proportion of inpatient resources utilized in the US. Their impact is growing within children's hospitals. Use of large administrative databases like the KID, by itself or in conjunction with other databases, may provide unique perspectives to answer the clinical questions raised by the study findings, including the following. (1) Does care quality differ for children with NI depending on the type of hospital they use? (2) Does inpatient resource utilization for children with NI vary depending on their access to medical homes and preventive care? And (3) How does substandard transition of health care services from pediatric to adult health care providers affect hospital use for adolescents and young adults with NI?

As a higher proportion of hospital resources are accounted for by children with NI over time, children's hospitals, in particular, will need to ensure that (1) adequate clinical and coordinated expertise is focused on the needs of these children, (2) NI clinical assessment and care management training is developed for trainees and junior graduates in pediatric postgraduate educational programs, (3) partnerships between families of children with NI and hospitals are developed and implemented, and (4) care treatment strategies of both nervous and non-nervous-system problems are rigorously evaluated for these children. These system-based efforts have the potential to promote continued quality of care for children with NI. We must ensure that the current health care system is staffed, educated, and equipped to serve, with efficiency and quality, this growing segment of vulnerable children.

## Supporting Information

Table S1 **Neurological impairment ICD-9-CM codes used in this study.**
(DOC)

## Abbreviations

CI: Confidence Interval
ICD-9-CM: *International Classification of Diseases, 9th Revision, Clinical Modification*
KID: Kids' Inpatient Database
MDC: Major Diagnostic Category
NI: neurological impairment
