# Supplementary material for: Trends in Resource Utilization by Children with Neurological Impairment in the United States Inpatient Health Care System: A Repeat Cross-Sectional Study
Source: PLoS Med. 2012 Jan 17;9(1):e1001158. doi: 10.1371/journal.pmed.1001158 (PMC3260313; doi:10.1371/journal.pmed.1001158)
Supplement: Table S1 — Neurological impairment ICD-9-CM codes used in this study. (DOC) [file pmed.1001158.s001.doc]

**Table S1: Neurological Impairment Categories, Specific Diseases and ICD-9-CM Diagnostic Codes**

| **Neurological Impairment Categories and Specific Diseases** | **ICD-9-CM Diagnostic Codes** |
| --- | --- |
| **Altered level of Consciousness** | |
| Central nervous system dysfunction in newborn not otherwise specified; Cerebral depression, coma, and other abnormal cerebral signs; Alteration of consciousness, Coma; Transient alteration of awareness; Persistent vegetative state | 7792, 78001, 78002, 78003 |
| **Anterior Horn Cell Disease** | |
| Werdnig-Hoffmann disease, Spinal muscular atrophy, Kugelberg-Welander disease, Progressive muscular atrophy, Progressive bulbar palsy, Pseudobulbar palsy, Primary lateral sclerosis, Amyotrophic lateral sclerosis | 3350, 3351, 3358, 3359, 33510, 33511, 33519, 33520, 33521, 33522, 33523, 33524, 33529 |
| **Asphyxia, Anoxia and Cardiac Arrest** | |
| Anoxic brain damage, Cardiac arrest*, Intrauterine hypoxia and birth asphyxia: Severe birth asphyxia, Mild or moderate birth asphyxia, Unspecified birth asphyxia in liveborn infant*; Asphyxia and hypoxemia, Drowning and nonfatal submersion*, Asphyxiation and strangulation | 3481, 4275*, 7685, 7686, 7689*, 79901, 9941*, 9947 |
| **Brain or Spinal Cord Abnormalities** | |
| Other spinocerebellar diseases; Syringomyelia and syringobulbia, Cerebral cysts*, Benign intracranial hypertension***,** Compression of brain, Cerebral edema, Hereditary hemorrhagic telangiectasia*, Anencephalus, Craniorachischisis, Iniencephaly, Encephalocele, Microcephalus*, Congenital reduction deformities of brain, Diastematomyelia, Hydromyelia, Pseudotumor Cerebri, Cerebral fungal calcification, Ataxia-telangiectasia, Coritsostriatal-spinal degeneration, Congenital cerebral cyst, Macroencephaly, Macrogyria, Megalencephaly, Multiple anomalies of brain NOS, Porencephaly, Ulegyria, Agenesis of nerve*, Displacement of brachial plexus*, Familial dysautonomia*, Jaw-winking syndrome*, Marcus-Gunn syndrome*, Riley-Day syndrome*, Amyelia*, Atelomyelia*, Congenital anomaly of spinal meninges*, Defective development of cauda equine*, Hypoplasia of spinal cord*, Myelatelia*, Myelodysplasia*, | 3348, 3360, 3480*, 3482*, 3484, 3485, 3488*, 3489*, 4480*, 7400, 7401, 7402, 7420, 7421*, 7422, 7424, 74251, 74253, 74259*, 7428*, 7429 |
| **Brain and Spinal Cord Neoplasm** | |
| Neoplasm of Brain, Cerebrum, Lobes, Ventricles (Choroid plexus  Floor of ventricle), Cerebellum (Cerebellopontine angle), Brain Stem, Meninges, Cranial Nerves, Spinal Cord, Cranial fossa not otherwise specified or other parts of the brain (Corpus callosum, Tapetum), Malignant neoplasm of contiguous or overlapping sites of brain whose point of origin cannot be determined, Benign neoplasm of the Brain*, Cranial Nerves (Olfactory bulb*), Meninges* or Spinal Cord*. | 1910, 1912, 1913, 1914, 1915, 1916, 1917, 1918, 1919, 1920, 1921, 1983, 2250*, 2251*, 2252*, 2253* |
| **Cerebral Laceration and Contusion** | |
| Cerebral cortex, cerebellar, brainstem or unspecified laceration or contusion, with or without mention of open intracranial wound with prolonged [more than 24 hours] loss of consciousness without return to pre-existing conscious level | 85105, 85115, 85125, 85135, 85145, 85165, 85175, 85185, 85195 |
| **Cerebral Palsy** | |
| Cerebral Palsy: monoplegic, diplegic; paraplegia, hemiplegic and quadriplegic; Cerebral palsy not otherwise specified | 3430, 3431, 3432, 3433, 3434, 3438, 3439 |
| **Cerebrovascular Disease Including Late Effects** | |
| Cerebrovascular disease;* Intracerebral hemorrhage of basilar, bulbar, cerebellar, cerebralcerebromeningeal, cortical, internal capsule, intrapontine, pontine, subcortical, ventricular); Rupture of blood vessel in brain*; Subarachnoid hemorrhage* (Meningeal hemorrhage, Ruptured berry aneurysm(congenital), cerebral aneurysm not otherwise specified); Other and unspecified intracranial hemorrhage*; Nontraumatic extradural hemorrhage*; Nontraumatic epidural hemorrhage; Subdural hemorrhage*; nontraumatic Subdural hematoma; Occlusion and stenosis of precerebral arteries (Basilar artery, Carotid artery, Vertebral artery, Multiple and bilateral, Other specified precerebral artery, Unspecified precerebral artery, Occlusion of cerebral arteries, Cerebral thrombosis, Cerebral embolism, Cerebral artery occlusion, unspecified; Transient cerebral ischemia (Basilar artery syndrome, Vertebral artery syndrome, Subclavian steal syndrome*, Vertebrobasilar artery syndrome, Other specified transient cerebral ischemias, Unspecified transient cerebral ischemia* (Impending cerebrovascular accident, Intermittent cerebral ischemia, Transient ischemic attack [TIA]); Acute, but ill-defined, cerebrovascular disease* (Apoplexy, cerebral seizure), Other and ill-defined cerebrovascular disease*, Cerebral atherosclerosis* (Atheroma of cerebral arteries, Cerebral arteriosclerosis); Other generalized ischemic cerebrovascular disease* [Acute cerebrovascular insufficiency, Cerebral ischemia (chronic) not otherwise specified], Hypertensive encephalopathy; Cerebral aneurysm, non-ruptured* (Internal carotid artery, intracranial portionInternal carotid artery not otherwise specified); Cerebral arteritis, Moyamoya disease, Nonpyogenic thrombosis of intracranial venous sinus*, Transient global amnesia; Other or unspecified cerebrovascular disease or lesion not otherwise specified*; Late effects of cerebrovascular disease* (cognitive deficits, aphasia, dysphasia, Hemiplegia/hemiparesis, monoplegia, other paralytic syndrome, alterations of sensations, disturbances of vision, apraxia, facial weakness, vertigo) | 430-438*, 430*, 431, 432*, 4320*, 4321*, 4329*, 433, 4330, 4331, 4332, 4333, 4338, 4339, 434, 4340, 4341, 4349, 435*, 4350, 4351, 4352*, 4353, 4358, 4359*, 436*, 437*, 4370*, 4371*, 4372, 4373*, 4374, 4375, 4376*, 4377, 4378*, 4379*, 438*, 4380, 4381, 43810, 43811, 43812, 43819, 4382, 43820, 43821, 43822, 4383, 43830, 43831, 43832, 4384, 43840, 43841, 43842, 4385, 43850, 43851, 43852, 43853, 4386*, 4387, 4388*, 43881, 43882, 43883, 43884, 43885, 43889, 4389, |
| **Concussion with late effects** | |
| Concussion with prolonged loss of consciousness, without return to pre-existing conscious level; Concussion, With loss of consciousness of unspecified duration | 8504, 8505 |
| **Drug Toxicity** | |
| Neuroleptic malignant syndrome | 33392 |
| **Encephalopathy** | |
| Other and unspecified cerebral irritability in newborn*, Reye's syndrome; Other conditions of brain; Encephalopathy, not elsewhere classified; Septic encephalopathy, Metabolic encephalopathy, Other encephalopathy; Kernicterus due to isoimmunization, Kernicterus not due to isoimmunization | 7791*, 33181, 3483, 34830, 34831, 34839, 7734, 7747 |
| **Epilepsy** | |
| Generalized non-convulsive epilepsy* (Absence seizures: atonic, typical), Generalized convulsive epilepsy (clonic, myoclonic, tonic, tonic-clonic, Grand mal), Petit mal status* (Epileptic absence status), Grand mal status* (Status epilepticus not otherwise specified), Partial epilepsy with impairment of consciousness (Epilepsy of the limbic system; partial secondarily generalized, with memory and ideational disturbances; psychomotor, psychosensory, temporal lobe, epileptic automatism); Localization-related (focal) (partial) epilepsy and epileptic syndromes with complex partial seizures, without mention of intractable epilepsy*; Epilepsy, other* [cursive (running) gelastic]; Epilepsy, unspecified* (epileptic convulsions, fits, or seizures NOS); Partial epilepsy without mention of impairment of consciousness; Infantile spasms (Hypsarrhythmia, Lightning spasms, Salaam attacks); Epilepsia partialis continua - Kojevnikov's epilepsy, Convulsions; Convulsions in newborn*; Convulsive disorder not otherwise specified, Fits not otherwise specified, Seizures not otherwise specified | 3450*, 34500*, 34501, 3451*, 34510*, 34511, 3452*, 3453*, 3454, 34540*, 34541, 3455*, 34550*, 34551, 3456, 34560, 34561, 3457, 34570, 34571, 3458, 34580*, 34581, 3459*, 34590*, 3489, 7790*, 7803, 78039 |
| **Fracture of Skull** | |
| Fracture of skull base*, fracture of skull vault*; Open or closed skull fracture (base, vault, face, multiple, other) with or without mention of intracranial injury, with prolonged [more than 24 hours] loss of consciousness, without return to pre-existing conscious level; Closed or open fracture with cerebral laceration and contusion, subarachnoid, subdural, or extradural or unspecified intracranial with prolonged [more than 24 hours] loss of consciousness, without return to pre-existing conscious level; Other and unqualified skull fractures* | 800*, 80005, 80015, 80025, 80035, 80045, 80055, 80065, 80075, 80085, 80095, 801*, 80105, 80115, 80125, 80135, 80145, 80155, 80165, 80175, 80185, 80195, 803*, 80305, 80315, 80325, 80335, 80345, 80355, 80365, 80375, 80385, 80395, 804*, 80405, 80415, 80425, 80435, 80445, 80455, 80465, 80475, 80485, 80495 |
| **Genetic Condtitions** | |
| Friedreich's ataxia, Hereditary spastic paraplegia, Other cerebellar ataxia- Cerebellar ataxia not otherwise specified, Spinocerebellar disease, unspecified; Down's syndrome, Patau's syndrome (Trisomy 13),  Edwards' syndrome (Trisomy 18), Chromosomal anomalies, Autosomal deletion syndromes*, Cri-du-chat syndrome*, Velo-cardio-facial syndrome*, Other microdeletions*, Other autosomal deletions*, Other conditions due to sex chromosome anomalies*, Other conditions due to chromosome anomalies, not elsewhere classified*, Other conditions due to autosomal anomalies*Accessory autosomes NEC, Klinefelter's syndrome* (XXY syndrome); Chromosomal anomalies, Other conditions due to chromosome anomalies*, Chromosomal anomalies, Conditions due to anomaly of unspecified chromosome*, Other and unspecified congenital anomalies (Congenital malformation syndromes affecting multiple systems, Laurence-Moon-Biedl syndrome), Multiple congenital anomalies, so described*, Other specified anomalies*, Prader-Willi syndrome, Marfan syndrome*, Fragile X syndrome, Other and unspecified congenital anomalies, Other specified anomalies, Other* | 3340, 3341, 3343, 3349, 7580, 7581, 7582, 7583*, 75831, 75832*, 75833*, 75839*, 75881*, 75889*, 7585*, 7587*, 7588*, 7589*, 7597*, 7598*, 75981, 74982*, 75983, 75989* |
| **Hydrocephalus** | |
| Communicating hydrocephalus*; Obstructive hydrocephalus (Acquired hydrocephalus NOS), Congenital hydrocephalus; Presence of cerebrospinal fluid drainage device [Cerebral ventricle (communicating) shunt, valve, or device in situ]* | 3313*, 3314, 7423, V452* |
| **Intracranial Hemorrhage or Injury** | |
| Subdural and cerebral hemorrhage [whether described as due to birth trauma or to intrapartum anoxia or hypoxia, Subdural hematoma (localized), or Tentorial tear], Intraventricular hemorrhage, Unspecified grade*, Intraventricular hemorrhage, Grade I* (Bleeding into germinal matrix), Intraventricular hemorrhage, Grade II* (Bleeding into ventricle), Intraventricular hemorrhage, Grade III (Bleeding with enlargement of ventricle), Intraventricular hemorrhage, Grade IV (Bleeding into cerebral cortex); Subarachnoid hemorrhage* (Subarachnoid hemorrhage from any perinatal cause); Subarachnoid, subdural, and extradural hemorrhage, following injury with open or closed intracranial wound with prolonged [more than 24 hours] loss of consciousness without return to pre-existing conscious level; Other and unspecified intracranial hemorrhage following injury, Without mention of open intracranial wound* (Cerebral compression due to injury, Intracranial hematoma following injury, Traumatic cerebral hemorrhage); Other and unspecified intracranial hemorrhage following injury, Without mention of open intracranial wound with prolonged [more than 24 hours] loss of consciousness without return to pre-existing conscious level; Intracranial injury of other and unspecified nature, With open intracranial wound* (Cerebral compression due to injury, Intracranial hematoma following injury; Traumatic cerebral hemorrhage); Intracranial injury of other and unspecified nature, With open intracranial wound with prolonged [more than 24 hours] loss of consciousness without return to pre-existing conscious level; Late effects of injuries to the nervous system, Late effect of intracranial injury without mention of skull fracture | 7670, 7721*, 77210*, 77211*, 77212*, 77213, 77214, 7722*, 8520, 85205, 85215, 85225, 85235, 85245, 85255, 8530*, 85305, 8541*, 85415, 9070 |
| **Leukodystrophies** | |
| Cerebral lipidoses [Amaurotic (familial) idiocy, Batten, Jansky-Bielschowsky, Kufs', Spielmeyer-Vogt, Tay-Sachs, Gangliosidosis]; Leukodystrophy (Krabbe's disease, Leukodystrophy not otherwise specified, globoid cell, metachromatic, sudanophilic, Pelizaeus-Merzbacher disease, Sulfatide lipidosis) | 3301, 3300 |
| **Meningitis, Encephalitis or Abscess*** | |
| Bacterial meningitis (Includes: arachnoiditis bacterial, leptomeningitis bacterial, meningitis bacterial, meningoencephalitis bacterial, meningomyelitis bacterial, pachymeningitis bacterial); Meningitis of unspecified cause or due to other organisms- (Includes: Arachnoiditis, leptomeningitis, meningitis, and pachymeningitis due to organisms other than bacteria); Encephalitis in viral diseases classified elsewhere [Code first underlying disease, as: cat-scratch disease (078.3), infectious mononucleosis (075), ornithosis (073.7)]; Encephalitis in rickettsial or protozoal diseases classified elsewhere; Other encephalitis due to infection classified elsewhere; Encephalitis following immunization procedures; Encephalopathy; Postinfectious encephalitis- [Infectious acute disseminated encephalomyelitis (ADEM)]; Toxic encephalitis [Code first underlying cause, as: carbon tetrachloride (982.1), hydroxyquinoline derivatives (961.3), lead (984.0-984.9), mercury (985.0), thallium (985.8)]; Noninfectious acute disseminated encephalomyelitis (ADEM); Intracranial and intraspinal abscess; Phlebitis and thrombophlebitis of intracranial venous sinuses; Late effects of intracranial abscess or pyogenic infection; Kernicterus; Reye’s Syndrome | 320, 321, 322, 3230, 3231, 3232, 3234, 3235, 3236, 3237, 3238, 33181, 3483, 34830, 34831, 34839, 7734, 7747, 7791, 3240, 3249, 325, 326, |
| **Mental Retardation** | |
| Mild mental retardation (High-grade defect IQ 50-70), Moderate mental retardation (IQ 35-49), Severe mental retardation (IQ 20-34), Profound mental retardation (IQ under 20), Unspecified mental retardation; Frontotemporal dementia; Dementia with Lewy bodies (Dementia with Parkinsonism, Lewy Body Dementia, Lewy Body disease), Mild cognitive impairment, so stated | 317, 3180, 3181, 3182, 319, 3311, 33119, 33182, 33183 |
| **Metabolic Disorders** | |
| Disorders of amino-acid transport and metabolism*; Disorders of amino-acid transport and metabolism, Disturbances of amino-acid transport* [Cystinosis, Cystinuria, Fanconi (-de Toni) (-Debré) syndrome, Glycinuria (renal), Hartnup disease], Disturbances of branched-chain amino-acid metabolism * (Disturbances of metabolism of leucine, isoleucine, and valine, Hypervalinemia,, Intermittent branched-chain ketonuria, Leucine-induced hypoglycemia, Leucinosis, Maple syrup urine disease);  Disturbances of sulphur-bearing amino-acid metabolism* (Cystathioninemia, Cystathioninuria, Disturbances of metabolism of methionine, homocystine, and cystathionine, Homocystinuria, Hypermethioninemia, Methioninemia); Disturbances of histidine metabolism* (Carnosinemia, Histidinemia, Hyperhistidinemia, Imidazole aminoaciduria), Disorders of urea cycle metabolism * (Argininosuccinic aciduria, Citrullinemia, Disorders of metabolism of ornithine, citrulline, argininosuccinic acid, arginine, and ammonia, Hyperammonemia Hyperornithinemia), Other disturbances of straight-chain amino-acid metabolism* [Glucoglycinuria, Glycinemia (with methylmalonic acidemia), Hyperglycinemia, Hyperlysinemia, Pipecolic acidemia, Saccharopinuria, Other disturbances of metabolism of glycine, threonine, serine, glutamine, and lysine]; Disorders of amino-acid transport and metabolism, Other specified disorders of amino-acid metabolism* (Alaninemia, Ethanolaminuria, Glycoprolinuria, Hydroxyprolinemia, Hyperprolinemia, Iminoacidopathy, Prolinemia, Prolinuria, Sarcosinemia); Unspecified disorder of amino-acid metabolism*;  Mucopolysaccharidosis* (Gargoylism, Hunter's syndrome, Hurler's syndrome, Lipochondrodystrophy, Maroteaux-Lamy syndrome, Morquio-Brailsford disease, Osteochondrodystrophy, Sanfilippo's syndrome, Scheie's syndrome); Other specified disorders of metabolism*; Primary carnitine deficiency; Carnitine deficiency due to inborn errors of metabolism*; Iatrogenic carnitine deficiency* (Carnitine deficiency due to: Hemodialysis, Valproic acid therapy), Other secondary carnitine deficiency*;  Disorders of mitochondrial metabolism* [Kearns-Sayre syndrome, Mitochondrial Encephalopathy, Lactic Acidosis and Stroke-like episodes (MELAS syndrome), Mitochondrial Neurogastrointestinal Encephalopathy syndrome (MNGIE), Myoclonus with Epilepsy and with Ragged Red Fibers (MERRF syndrome), Neuropathy, Ataxia and Retinitis Pigmentosa (NARP syndrome)];  Other specified disorders of metabolism* [Hand-Schüller-Christian disease, Histiocytosis (acute) (chronic), Histiocytosis X (chronic), Excludes: histiocytosis: acute differentiated progressive (202.5) X, acute (progressive) (202.5)];  Phenylketonuria [PKU]- Hyperphenylalaninemia; Other disturbances of aromatic amino-acid metabolism; Lipidoses; Other disorders of purine and pyrimidine metabolism;  Disorders of fatty acid oxidation [Carnitine palmitoyltransferase deficiencies (CPT1, CPT2), Glutaric aciduria type II (type IIA, IIB, IIC)Long chain 3-hydroxyacyl CoA dehydrogenase deficiency (LCHAD)  Long chain/very long chain acyl CoA dehydrogenase deficiency (LCAD, VLCAD), Medium chain acyl CoA dehydrogenase deficiency (MCAD), Short chain acyl CoA dehydrogenase deficiency (SCAD)  Excludes: primary carnitine deficiency (277.81)]  Peroxisomal disorders [Adrenomyeloneuropathy, Neonatal adrenoleukodystrophy, Rhizomelic chrondrodysplasia punctata, X-linked adrenoleukodystrophy, Zellweger syndrome] | 270*, 2700*, 2703*, 2704*, 2705*, 2706*, 2707*, 2708*, 2709*, 2775*, 2778*, 27782*, 27783*, 27784*, 27787*, 27789*, 2701, 2702, 2727, 2772, 27781, 27785, 27786 |
| **Movement Disorders** | |
| Parkinson's disease, Paralysis agitans (Parkinsonism or Parkinson's disease: NOS, idiopathic, primary); Parkinson's disease, Secondary Parkinsonism (Neuroleptic-induced Parkinsonism, Parkinsonism due to drugs); Other degenerative diseases of the basal ganglia (Atrophy or degeneration: olivopontocerebellar [Déjérine-Thomas syndrome], pigmentary pallidal [Hallervorden-Spatz disease] striatonigral Parkinsonian syndrome associated with: idiopathic orthostatic hypotension, symptomatic orthostatic hypotension, Progressive supranuclear ophthalmoplegia, Shy-Drager syndrome); Myoclonus (Familial essential myoclonus, Progressive myoclonic epilepsy, Unverricht-Lundborg disease); Huntington's chorea; Other choreas (Hemiballism(us), Paroxysmal choreo-athetosis); Idiopathic torsion dystonia (Dystonia: deformans progressive,  musculorum deformans, Ziehen-Oppenheim disease), Symptomatic torsion dystonia (Athetoid cerebral palsy [Vogt's disease]), Double athetosis syndrome, Neuroleptic-induced acute dystonia); Other and unspecified extrapyramidal diseases and abnormal movement disorders; Cerebellar ataxia in diseases classified elsewhere; | 3320, 3321, 3330, 3332, 3334, 3335, 3336, 3337, 3339, 33390, 3344 |
| **Muscular Dystrophies and Myopathies** | |
| Congenital hereditary muscular dystrophy (Benign congenital myopathy), Central core disease, Centronuclear myopathy, Myotubular myopathy, Nemaline body disease , Hereditary progressive muscular dystrophy  (Muscular dystrophy: not otherwise specified, distal, Duchenne, Erb's, fascioscapulohumeral, Gower's, Landouzy-Déjérine, limb-girdle, ocular, oculopharyngeal); Myotonic disorders (Dystrophia myotonica, Eulenburg's disease, Myotonia congenital, Paramyotonia congenital, Steinert's disease, Thomsen's disease);  Familial periodic paralysis (Hypokalemic familial periodic paralysis), Toxic myopathy, Myopathy in endocrine diseases, Symptomatic inflammatory myopathy, Critical illness myopathy, Other Myopathies* [Acute necrotizing myopathy, Acute quadriplegic myopathy, Intensive care (ICU) myopathy, Myopathy of critical illness] | 3590, 3591, 3592, 3593, 3594, 3595, 3596, 3598*, 35981, 35989, 3599, |
| **Myelopathy or Myelitis** | |
| Vascular myelopathies (Acute infarction of spinal cord (embolic) (nonembolic), Arterial thrombosis of spinal cord, Edema of spinal cord), Hematomyelia, Subacute necrotic myelopathy , Subacute combined degeneration of spinal cord, Other myelopathy* (drug induced, radiation induced); Multiple sclerosis, Neuromyelitis optica, Schilder's disease (Balo's concentric sclerosis, Encephalitis periaxialis: concentrica [Balo's], diffusa [Schilder's]), Acute (transverse) myelitis, Idiopathic transverse myelitis, De-myelinating disease of central nervous system* (Central demyelination of corpus callosum, Central pontine myelinosis); Marchiafava (-Bignami) disease , unspecified* (Disseminated or multiple sclerosis: not otherwise specified, brain stem, cord, generalized), Myasthenia Gravis* (Myasthenia gravis in crisis), Toxic myoneural disorders*, unspecified*- Cord compression; Hemiplegia and hemiparesis | 3361, 3362, 3363, 3368, 3369  340, 3410, 3411, 34120, 34121, 34122, 3418*, 3419*, 3420, 3421, 3428, 3429, 3580*, 35800, 35801, 3581, 3582, 3588*, 3589* |
| **Neurodegenerative** | |
| Cerebral degeneration in generalized lipidoses (Fabry's disease, Gaucher's disease, Niemann-Pick disease, sphingolipidosis); Other specified cerebral degenerations in childhood (Hunter's disease, Mucopolysaccharidosis); Unspecified cerebral degeneration in childhood (Alpers' disease or gray-matter degeneration, Infantile necrotizing encephalomyelopathy, Leigh's disease, Subacute necrotizing encephalopathy or encephalomyelopathy, Stiffman syndrome); Other frontotemporal dementia; Cerebral degeneration in diseases classified elsewhere; Other cerebral degeneration; Primary cerebellar degeneration (Cerebellar ataxia: Marie's, Sanger-Brown, Dyssynergia cerebellaris myoclonica, Primary cerebellar degeneration: not otherwise specified, hereditary, sporadic) | 3302, 3303, 3308, 3309, 3317, 33189, 33391, 3342 |
| **Optic Atrophy and other Visual Abnormalities** | |
| Blindness and low vision, Profound impairment, both eyes, level not further specified; Optic atrophy; Optic neuritis; Other disorders of optic nerve (Ischemic optic neuropathy, Hemorrhage in optic nerve sheaths, Other - Compression of optic nerve); Disorders of optic chiasm, visual pathway or visual cortex (associated with pituitary neoplasms, vascular disorders, inflammatory disorders); Cortical Blindness; Paralytic strabismus* (Paralytic strabismus, unspecified; Third or oculomotor nerve palsy, partial; Third or oculomotor nerve palsy, total; Fourth or trochlear nerve palsy; Sixth or abducens nerve palsy; External ophthalmoplegia; Total ophthalmoplegia); Other specified strabismus (Duane's syndrome, Progressive external ophthalmoplegia, Strabismus in other neuromuscular disorders); Other disorders of binocular eye movements (Palsy of conjugate gaze, Spasm of conjugate gaze, Convergence insufficiency or palsy, Convergence excess or spasm, Anomalies of divergence, Internuclear ophthalmoplegia, Other dissociated deviation of eye movements); Unspecified disorder of eye movements* (Ophthalmoplegia or strabismus not otherwise specified); Nystagmus and other irregular eye movements*; Nystagmus, unspecified*; Congenital nystagmus*; Latent nystagmus*; Visual deprivation nystagmus*; Nystagmus associated with disorders of the vestibular system*; Dissociated nystagmus*; Other forms of nystagmus*; Deficiencies of saccadic eye movements* - Abnormal optokinetic response; Deficiencies of smooth pursuit movements*; Other irregularities of eye movements*- Opsoclonus  Anophthalmos; Clinical anophthalmos, unspecified – Agenesis (Congenital absence of eye), Anophthalmos NOS; Cystic eyeball, congenital; Cryptophthalmos; Microphthalmos; Microphthalmos, unspecified; Simple microphthalmos; Microphthalmos associated with other anomalies of eye and adnexa; Buphthalmos  Buphthalmos, unspecified; Simple buphthalmos; Buphthalmos associated with other ocular anomalies (Keratoglobus, congenital, associated with buphthalmos, Megalocornea associated with buphthalmos); Congenital cataract and lens anomalies; Congenital cataract, unspecified; Capsular and subcapsular cataract  Cortical and zonular cataract; Nuclear cataract; Total and subtotal cataract, congenital; Congenital aphakia  Anomalies of lens shape; Congenital ectopic lens; Congenital cataract and lens anomalies, Other  Coloboma and other anomalies of anterior segment; Anomalies of corneal size and shape; Corneal opacities, interfering with vision, congenital; Other corneal opacities, congenital; Specified anomalies of anterior chamber, chamber angle, and related structures; Aniridia; Other specified anomalies of iris and ciliary body  Specified anomalies of sclera; Multiple and combined anomalies of anterior segment; Coloboma and other anomalies of anterior segment, Other Congenital anomalies of posterior segment; Vitreous anomalies- Congenital vitreous opacity; Fundus coloboma; Chorioretinal degeneration, congenital; Congenital folds and cysts of posterior segment; Congenital macular changes; Other retinal changes, congenital; Specified anomalies of optic disc - Coloboma of optic disc (congenital); Vascular anomalies - Congenital retinal aneurysm; | 36900, 3771, 37710, 37711, 37712, 37713, 37714, 37715, 37716, 3773, 37730, 37731, 37732, 37733, 37734, 37739, 3774, 37741, 37742, 37749, 3775, 37751, 37752, 37753, 37754, 3776, 37761, 37762, 37763, 3777, 37771, 37772, 37773, 37775, 3785, 37850, 37851, 37852, 37853, 37854, 37855, 37856, 3787*, 37871, 37872, 37873, 3788,  37881, 37882, 37883, 37884, 37885, 37886, 37887, 3789*, 3795*, 37950*, 37951*, 37952*, 37953*, 37954*, 37955*, 37956*, 37957*, 37958, 37959*, 7430, 74300, 74303, 74306, 7431, 74310, 74311, 75312, 7432, 74320, 74321, 74322, 7433, 74330, 74331, 74332, 74333, 74334, 74335, 74336, 74337, 74339, 7434, 74341, 74342, 74343, 74344, 74345, 74346, 74347, 74348, 74349, 7435, 74351, 74352, 74353, 74354, 74355, 74356, 74357, 74358, 74359 |
| **Other Paralytic Syndromes** | |
| Quadriplegia and quadriparesis; Quadriplegia, unspecified (C_1_-C_4_, complete, Quadriplegia, C1-C4, incomplete, C_5_-C_7_, complete, Quadriplegia, C5-C7, incomplete); Other quadriplegia; Paraplegia; Diplegia of upper limbs;  Monoplegia of upper or lower limb; Unspecified monoplegia; Cauda equina syndrome without mention of neurogenic bladder; Cauda equina syndrome with neurogenic bladder; Locked-in state; Other specified paralytic syndromes  Paralysis, unspecified | 3440, 34400, 34401, 34402, 34403, 34404, 34409, 3441, 3442, 3443, 34430, 34431, 34432, 3444, 34440, 34441, 34442, 3445, 34460, 34461, 34481, 34489, 3449 |
| **Peripheral Nervous System Abnormality** | |
| Trigeminal nerve disorders* (disorders of 5th cranial nerve), Trigeminal neuralgia* (Tic douloureux, Trifacial neuralgia, Trigeminal neuralgia not otherwise specified), Atypical face pain*, Facial nerve disorders* (Bell's palsy*- Facial palsy, Geniculate ganglionitis*), Disorders of other cranial nerves* (Disorders of olfactory [1st] nerve*, Glossopharyngeal neuralgia*, Other disorders of glossopharyngeal [9th] nerve*, Disorders of pneumogastric [10th] nerve* Disorders of vagal nerve, Disorders of accessory [11th] nerve*, Disorders of hypoglossal [12th] nerve*, Multiple cranial nerve palsies* (Collet-Sicard syndrome, Polyneuritis cranialis); Unspecified disorder of cranial nerves, Nerve root and plexus disorders* (Brachial plexus lesions*, Lumbosacral plexus lesions*)  Cervical root lesions not elsewhere classified*, Thoracic root lesions, not elsewhere classified*, Lumbosacral root lesions not elsewhere classified*, Neuralgic amyotrophy, Phantom limb (syndrome), Other nerve root and plexus disorders, Carpal tunnel syndrome - Median nerve entrapment, Partial thenar atrophy; Other lesion of median nerve - Median nerve neuritis, Lesion of ulnar nerve -Cubital tunnel syndrome, Tardy ulnar nerve palsy, Lesion of radial nerve - Acute radial nerve palsy;  Mononeuritis multiplex; Carpal tunnel syndrome; Other mononeuritis of upper limb, Mononeuritis of upper limb, unspecified; Mononeuritis of lower limb (Lesion of sciatic nerve), Meralgia paresthetica*- Lateral cutaneous femoral nerve of thigh compression or syndrome; Other lesion of femoral nerve; Lesion of lateral popliteal nerve - Lesion of common peroneal nerve; Lesion of medial popliteal nerve; Tarsal tunnel syndrome; Lesion of plantar nerve - Morton's metatarsalgia, neuralgia, or neuroma; Causalgia of lower limb;    Hereditary and idiopathic peripheral neuropathy (Hereditary peripheral neuropathy - Déjérine-Sottas disease), Peroneal muscular atrophy - Charcot-Marie-Tooth disease; Neuropathic muscular atrophy, Hereditary sensory neuropathy, Refsum's disease (Heredopathia atactica polyneuritiformis), Idiopathic progressive polyneuropathy, Other specified idiopathic peripheral neuropathy (Supranuclear paralysis), Unspecified, Inflammatory and toxic neuropathy* (Acute infective polyneuritis*- Guillain-Barre syndrome, Postinfectious polyneuritis);  Polyneuropathy in collagen vascular disease (Code first underlying disease, as disseminated lupus erythematosus, polyarteritis nodosa, rheumatoid arthritis), Polyneuropathy in diabetes; Polyneuropathy in malignant disease; Polyneuropathy in other diseases classified elsewhere (Code first underlying disease, as amyloidosis, beriberi, deficiency of B vitamins, diphtheria, hypoglycemia, pellagra, porphyria, sarcoidosis, uremia) Alcoholic polyneuropathy*, Polyneuropathy due to drugs*, Polyneuropathy due to other toxic agents*, Inflammatory and toxic neuropathy* (Chronic inflammatory demyelinating polyneuritis*, Critical illness polyneuropathy*, Other inflammatory and toxic neuropathy*) | 350*, 3501*, 3502*, 3508*, 3509*, 351*, 3510*, 3511*, 3518*, 3519*, 352*, 3520*, 3521*, 3522*, 3523*, 3524*, 3525*, 3526*, 3529*, 353*, 3530*, 3531*, 3532*, 3533*, 3534*, 3538*, 3539*, 3551*, 3569*, 357*, 3570*, 3574*, 3576*, 3577*, 35782*,  3535, 3536, 354, 3540, 3541, 3542, 3543, 3544, 3545, 3548, 3549, 355, 3550, 3552, 3553, 3554, 3555, 3556, 3557, 35571, 35579, 3558, 3559, 356, 3560, 3561, 3562, 3563  3564, 3568, 3571, 3572, 3573, 3575, 3578, 35781, 35789 |
| **Pervasive Developmental Disorder** | |
| Pervasive developmental disorders; Autistic disorder; Childhood disintegrative disorder; Other specified pervasive developmental disorders (Asperger's disorder, Atypical childhood psychosis, Borderline psychosis of childhood); Unspecified pervasive developmental disorder (mild psychosis NOS, Pervasive developmental disorder NOS, Schizophrenia, childhood type NOS, Schizophrenic syndrome of childhood NOS) | 299, 2990, 2991, 2998, 2999 |
| **Prenatal Brain Injury** | |
| Congenital rubella; Congenital cytomegalovirus infection; Other congenital infections* (Congenital: herpes simplex, listeriosis, malaria, toxoplasmosis, tuberculosis); Other and ill-defined conditions originating in the perinatal period, Periventricular leukomalacia | 7710, 7711, 7712*, 7797 |
| **Spina Bifida** | |
| Spina bifida, with or without hydrocephalus (Arnold-Chiari syndrome, type II) cervical, dorsal (thoracic), lumbar or unspecified region; Hydromeningocele (spinal), Hydromyelocele, Meningocele (spinal), Meningomyelocele, Myelocele, Myelocystocele, Rachischisis,Spina bifida (aperta), Syringomyelocele; | 7410, 74100, 74101, 74102, 74103, 7419, 74190, 74191,  74192, 74193 |
| **Tuberous Sclerosis** | |
| Tuberous sclerosis (Bourneville's disease, Epiloia), Other hamartoses [Peutz-Jeghers, Sturge-Weber (Dimitri), von Hippel-Lindau] | 7595, 7596 |

* indicates an ICD-9-CM code evaluated as “maybe” by one or more pediatric neurologists during the code review process.
